# Molecular Mechanisms of PD-1 and PD-L1 Activity on a Pan-Cancer Basis: A Bioinformatic Exploratory Study

**DOI:** 10.3390/ijms22115478

**Published:** 2021-05-22

**Authors:** Siddarth Kannan, Geraldine Martina O’Connor, Emyr Yosef Bakker

**Affiliations:** School of Medicine, University of Central Lancashire, Preston PR1 2HE, UK; SKannan@uclan.ac.uk

**Keywords:** immunotherapy, immune checkpoint blockade, cBioPortal, bioinformatics, computational biology, survival analysis, OncomiR, microRNAs

## Abstract

Immune checkpoint blockade targeting PD-1 (PDCD1)/PD-L1 (CD274) is increasingly used for multiple cancers. However, efficacy and adverse-related events vary significantly. This bioinformatic study interrogated molecular differences pertaining to PDCD1/CD274 and their correlated genes on a pan-cancer basis to identify differences between cancer types. Patient RNA-seq data from fifteen cancer types were accessed on cBioPortal to determine the role of PDCD1/CD274 in patient survival and to identify positively and negatively correlated genes, which were also assessed for clinical relevance. Genes correlating with PDCD1/CD274 across multiple cancers were taken forward for drug repurposing via DRUGSURV and microRNA analysis using miRDB and miRabel. MicroRNAs were also screened for clinical relevance using OncomiR. Forty genes were consistently correlated with PDCD1/CD274 across multiple cancers, with the cancers themselves exhibiting a differential role for the correlated genes in terms of patient survival. Esophageal and renal cancers in particular stood out in this regard as having a unique survival profile. Forty-nine putative microRNAs were identified as being linked to the PDCD1/CD274 network, which were taken forward and further assessed for clinical relevance using OncomiR and previously published literature. One hundred and thirty significant survival associations for 46 microRNAs across fourteen groups of cancers were identified. Finally, a total of 23 putative repurposed drugs targeting multiple components of the PDCD1/CD274 network were identified, which may represent immunotherapeutic adjuvants. Taken together, these results shed light on the varying PDCD1/CD274 networks between individual cancers and signpost a need for more cancer-specific investigations and treatments.

## 1. Introduction

While cancer immunotherapy has a rich and interesting history [1], recent advances in our understanding of the complex interactions between the tumor and immune cells have provided the opportunity to exploit the power of the immune system for the benefit of patients. A range of different approaches have been explored in the past including the use of cytokines, anti-tumor antibodies, adoptive cell transfer, and cancer vaccines, with the use immune checkpoint blockade agents coming to prominence over the last decade.

Immune checkpoint molecules include cytotoxic T lymphocyte-associated protein 4 (CTLA-4) and programmed cell death protein 1 (PDCD1, PD-1). Both molecules act to inhibit T lymphocytes—CTLA-4 acts to block T cell co-stimulatory signaling via preventing the interactions between CD28 and CD80/86, and PD-1 via T cell inhibitory signaling upon engagement with its ligands PD-L1 (CD274) and PD-L2 (PDCD1LG2). Strategies that block these T cell-inhibitory molecules have proved successful in unleashing the patient’s own T cell immunity against tumor antigens in cancer. 

PDCD1 was first described in 1992 [2] and analysis of knockout mouse models revealed the role it plays in suppressing T cell responses with the development of autoimmunity in its absence [3,4]. Expression of CD274 ligands on tumor cells was found to contribute to tumor immune evasion and, in mouse models, antibody-mediated blockade of CD274 caused suppression of transplanted melanoma tumor growth [5]. Based on these observations nivolumab, a fully humanized anti-PDCD1 antibody, was developed and approved for treatment, initially for malignant melanoma. Since then, a variety of other agents have been developed including those targeting CD274 (atezolizumab, avelumab, and durvalumab) as well as PDCD1 (pembrolizumab and cemiplimab). The range of approved cancers has also grown to include small and non-small cell lung cancer, renal cell carcinoma, squamous cell head and neck carcinoma, non-Hodgkin lymphoma, gastric cancer, triple negative breast cancer, and cervical cancer, among others (reviewed in [6]).

Recent meta-analyses of the efficacy of antibodies targeting PDCD1/CD274 as monotherapy [7,8] found that this treatment is associated with more tumor responses and increased overall survival (OS) compared to conventional therapy. Although there are some patients who show long-term complete responses [9,10], the average response rate is 20% with significant differences across different tumor types [8]. It is of significant interest to identify the minority of patients who will benefit from immune checkpoint blockade to better direct this therapy and avoid the (often immune-mediated) adverse events [11]. Higher objective response rates to PDCD1/CD274 monotherapy have been associated with a number of factors, including immunogenicity of the tumor [8], male sex, age < 65 years, current and former smokers, a lack of central nervous system or liver metastasis, and a lack of EGFR mutations [7]. Therapy responses are not limited to those cancers in which CD274 expression is detected but high CD274 is associated with a greater response [8,12,13]. In contrast, high levels of serum [14] or exosomal [15] CD274 have been associated with non-responders.

One approach to increase the pool of patients who benefit from checkpoint blockade therapy is to explore combination therapy. A recent analysis of clinical trials found that PDCD1/CD274 monotherapy studies are declining from a peak in 2017, while combination therapies show consistent increases over time and represent a large majority of recent trials [16]. A range of different agents have been explored in combination, including chemotherapy and interventions targeting CTLA-4 and VEGF [16]. An early study in melanoma found that the combination of the anti-CTLA-4 antibody ipilimumab plus nivolumab (anti-PD-1) resulted in responses greater than those seen in monotherapy trials [17]. At maximum acceptable doses, objective responses were observed in 53% of patients, with responding patients showing tumor reduction of at least 80% [17]. Identification of appropriate adjuvant therapies is of considerable interest, with the repositioning of previously approved drugs potentially minimizing the cost and time needed for clinical use approval [18].

This exploratory study uses a bioinformatic approach to examine the impact of mRNA levels of PDCD1, CD274, and genes showing correlated expression levels on the survival outcome of a range of cancers. Based on a panel of genes predicted to impact patient outcomes, potential therapeutic strategies were explored including the prediction of microRNAs and identification of approved drugs capable of targeting the relevant proteins. In this way, additional therapeutic targets, likely to complement direct PDCD1/CD274 antibody targeting, are identified.

## 2. Results

### 2.1. Impact of CD274 and PDCD1 Expression on Cancer Patient Survival

**Figure 1:**
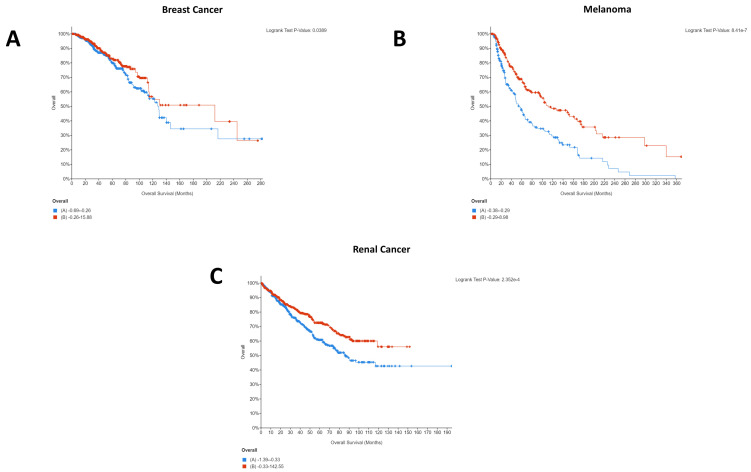
Statistically significant Kaplan–Meier plots for pan-cancer CD274 expression. In all cases, overall survival in months can be seen on the *x*-axis whilst percentage survived is shown on the *y*-axis. In all cases, high CD274 expression is shown in red whilst low CD274 expression is shown in blue. Data were generated using the mRNA expression z-scores relative to diploid samples (RNA Seq V2 RSEM) for the studies described in the Materials and Methods. CD274 expression was a significant factor for patient survival only in breast cancer (**A**), melanoma (**B**), and renal cancer (**C**). As such, bladder, cervical, colorectal, esophageal, gastric, head and neck, liver, lung, lymphoma, mesothelioma, ovarian, and pancreatic cancers have not been shown here but are available in Figure S1. Additionally, the mesothelioma study included (Mesothelioma (TCGA, Firehose Legacy)) lacked usable Kaplan–Meier survival data.

To assess the importance of CD274 expression on patient survival, the cBioPortal database was accessed due to its wealth of patient-level omics data [19,20]. Data selection was performed on studies containing “mRNA expression z-scores relative to diploid samples (RNA Seq V2 RSEM)” analysis, as described in the Materials and Methods. Individual studies were collated on a per-cancer type basis and patients were split by the median CD274 expression into high (red) and low (blue) groups on the Kaplan–Meier curves below in Figure 1:

Only melanoma, breast, and renal cancers demonstrated a statistically significant difference in patient survival between the low and high expression groups. Other cancers, detailed in the legend of Figure 1, have thus been excluded from the graph but can be seen in Figure S1. Strikingly, for the patient cohorts and cancer types selected, high CD274 expression appeared to be beneficial for breast cancer (Figure 1A), melanoma (Figure 1B), and renal cancer (Figure 1C) patients.

**Figure 2:**
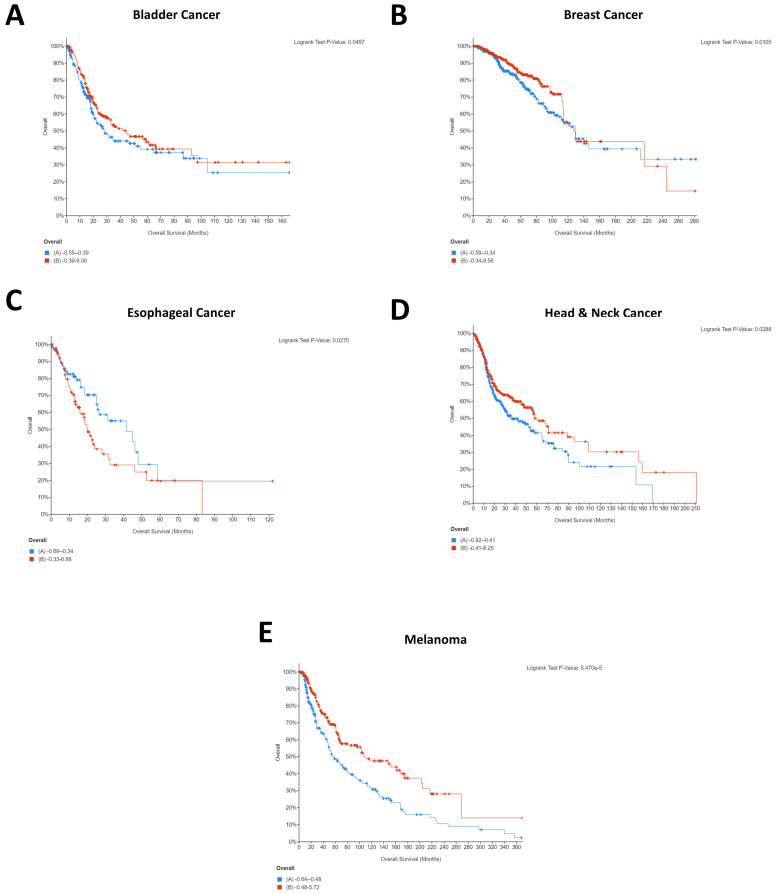
Statistically significant Kaplan–Meier plots for pan-cancer PDCD1 expression. In all cases, overall survival in months can be seen on the *x*-axis whilst percentage survived is shown on the *y*-axis. In all cases, high PDCD1 expression is shown in red whilst low PDCD1 expression is shown in blue. Data were generated using the mRNA expression z-scores relative to diploid samples (RNA Seq V2 RSEM) for the studies described in the Materials and Methods. PDCD1 expression was a significant factor for patient survival only in bladder cancer (**A**), breast cancer (**B**), esophageal cancer (**C**), head and neck cancer (**D**), and melanoma (**E**). As such, cervical, colorectal, gastric, liver, lung, lymphoma, mesothelioma, ovarian, renal, and pancreatic cancers have not been shown here but are available in Figure S2. Additionally, the mesothelioma study included (Mesothelioma (TCGA, Firehose Legacy)) lacked usable Kaplan–Meier survival data.

In addition to CD274, analysis of PDCD1 expression was also performed. Although PDCD1 most typically relates to its expression on tumor-infiltrating lymphocytes (TILs), there is increasing evidence of tumor-intrinsic PDCD1 expression. In the case of melanoma, for example, tumor-intrinsic PDCD1 expression exhibits an oncogenic effect, whereas in lung cancer the blockade of tumor-intrinsic PDCD1 expression promoted proliferation [21]. As such, to further understand the impact of PDCD1 between different cancer types, it is essential to also consider PDCD1 expression, as shown below in Figure 2:

Interestingly, several cancers which did not show significant effects for CD274 for the patient cohorts selected demonstrated significant impact of PDCD1 expression on patient survival, including bladder cancer, esophageal cancer, and head and neck cancer. Conversely, renal cancer demonstrated a significant effect of CD274 expression on patient survival (Figure 1C) but did not show an effect of PDCD1. Melanoma and breast cancer both demonstrated that high expression of CD274 was significantly associated with patient survival (Figure 1A,B) and showed largely the same pattern for PDCD1 (Figure 2B,E). Of the five cancers that demonstrated a significant role for PDCD1 in patient survival, esophageal cancer stood out with low PDCD1 expression being beneficial for the patient cohort (Figure 2C), whereas the other four cancers all showed high PDCD1 expression to be beneficial (Figure 2A,B,D,E). The difference in clinical relevance of CD274 and PDCD1 points to the unique molecular biology of each individual cancer type and highlights a need to identify where the differences lie. This indicates the individuality of tumor immunogenic responses and prompts a need for further molecular insight.

### 2.2. Identification of Genes That Correlate with PDCD1 and CD274 Pan-Cancer 

To delineate potential molecular mechanisms surrounding the differing clinical relevance of PDCD1/CD274, co-expression analysis was undertaken. Using the same studies as in the previous section per cancer type, co-expressed genes with a cut-off of ≥0.6 or ≤−0.6 (Spearman’s correlation coefficient) were identified using cBioPortal [19,20]. After following the filtering process described in the Materials and Methods, the genes shown below in Table 1 were identified as being significantly co-expressed with CD274 or PDCD1 in multiple cancers:

Although positively correlated and negatively correlated genes were identified for CD274, there were no genes negatively correlated with PDCD1 that remained following the filtration process described in the Materials and Methods. Therefore, only positively correlated genes are shown for PDCD1.

### 2.3. Assessing Clinical Relevance of Co-Expressed Genes

After identifying the co-expressed genes to be taken forward (Table 1), genes underwent Kaplan–Meier survival analysis using the same studies and approach as described previously. Table 2 below summarizes the results obtained for the CD274-correlated genes. 

It should be noted that although CCR5 produced a statistically significant Kaplan–Meier plot for breast cancer, it has not been included in the table due to its expression status impacting patient survival differentially across different time points. For the first 120 months, high expression was evidently beneficial, but this effect flipped after this timepoint.

Strikingly, it is immediately apparent that there is a series of cancer-specific gene associations with survival for both the genes that were found to be positively associated with CD274 (CCR5, CD80, GBP1, GBP5, JAK2, LCP2, PDCD1LG2, and SAMD9L) and those showing a negative correlation (COX19, DNASE1, DUS1L, and GATD3A). For example, DUS1L, a gene negatively correlated with CD274, was shown in ovarian, gastric, and breast cancer to be beneficial at high levels of expression. This contrasts with the finding in melanoma and renal cancer, where low expression was beneficial for patients. Similarly, low expression of SAMD9L, positively correlated with CD274, was beneficial for lymphoma patients, whereas high expression of the same gene was beneficial for melanoma and breast cancer patients. These differences may provide insight into the differing molecular networks underlying the response to immunotherapy.

The same analysis was performed for PDCD1-correlated genes, though, as stated, there were no negatively correlated genes taken forward at this stage of the analysis. Table 3 below summarizes the data:

Similar to the striking differences observed with the CD274-correlated genes, there are again some differences with the PDCD1-correlated genes that are immediately apparent between cancers. Renal cancer in particular stands out, with the majority of the genes demonstrating low expression to be beneficial for patients. Comparatively, high expression of the same genes was shown to be beneficial in the majority of the other cancer types. Similarly, genes that were significantly associated with survival in the majority of cancers had no such effect in esophageal cancer. These potential differences could go some way to explaining the differing role that PDCD1 expression has on patient survival, given that esophageal cancer was distinct from the other four cancers in that low PDCD1 was beneficial for esophageal cancer patients (Figure 2C). This again highlights the cancer-specific networks that may exist and signposts a need for delineation of analysis for individual types of cancer. 

### 2.4. Identification of microRNAs Targeting CD274, PDCD1, and Their Correlated Genes

To further elucidate potential mechanisms surrounding CD274/PDCD1 and their correlated genes, the miRDB microRNA database [22] and the miRabel microRNA database [23] were used to identify potential microRNAs that target the genes of interest. Using a score of ≥80 as the threshold for miRDB and a score of ≤0.05 for miRabel, microRNAs were collated for CD274, PDCD1, and their correlated genes. Following the filtering process described in the Materials and Methods, a total of 49 unique microRNAs were identified that appeared in both databases, shown below in Table 4:

### 2.5. Assessing Clinical Relevance of the microRNAs Targeting CD274/PDCD1-Related Genes

**Figure 3:**
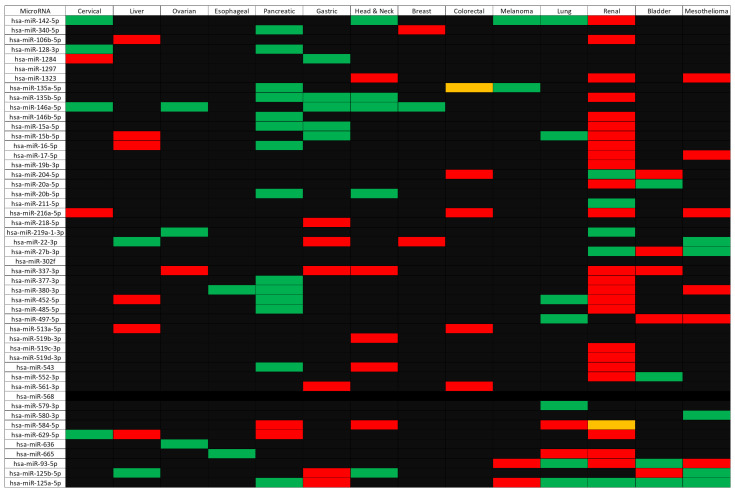
Heatmap of the clinical relevance of the microRNAs from Table 4. MicroRNAs are shown on the *y*-axis whilst cancer types are shown on the *x*-axis. A green color indicates that high expression of the microRNA is significantly beneficial for that cancer type (*p* ≤ 0.05), whilst red indicates that low expression of the microRNA is significantly beneficial for survival (*p* ≤ 0.05). Orange indicates mixed effects, whilst black indicates no significant relationship (*p* > 0.05) or, in the case of hsa-miR-568, no output data from OncomiR [24].

After identifying the microRNAs described above, they were then screened for their importance in patient survival using OncomiR [24]. The findings from OncomiR are summarized in the heatmap (Figure 3) below:

Overall, Figure 3 above summarizes the primary finding of the OncomiR analysis, which is the identification of 130 significant survival associations for 46 microRNAs across fourteen groups of cancer. Individual survival results can be seen in File S5. No microRNA selected was significantly associated with survival in lymphoma, hence its exclusion from the heatmap. Although the vast majority of microRNAs showed effects which are evidently clear in Figure 3, hsa-miR-135a-5p and hsa-miR-584-5p showed multiple effects on colorectal and renal cancer respectively. Low expression of hsa-miR-135a-5p was significantly beneficial for colon cancer patients, whilst high expression of hsa-miR-135a-5p was significantly beneficial for rectal cancer patients. Interestingly, hsa-miR-584-5p demonstrated mixed effects on renal cancers, with high expression being significantly beneficial for kidney renal clear cell carcinoma patients, whereas low expression was significantly beneficial for kidney renal papillary cell carcinoma patients.

Another pattern that immediately becomes apparent is the trend for renal cancer to exhibit opposite survival relationships to other cancer types. Similar to the Kaplan–Meier survival analysis of CD274/PDCD1-correlated genes (Table 2 and Table 3), many of the microRNAs showed an opposite effect on survival for renal cancer patients compared to other cancer types, e.g., high expression of hsa-miR-142-5p was beneficial in cervical cancer, head and neck cancer, melanoma, and lung cancer, but low expression of the same microRNA was beneficial in renal cancer. Similarly, high expression of hsa-miR-146b-5p, hsa-miR-15a-5p, hsa-miR-16-5p, and hsa-miR-377-3p was beneficial for pancreatic cancer patients, whereas low expression of the same microRNAs was beneficial for renal cancer patients. There are numerous trends and findings to be seen in the OncomiR survival analysis in Figure 3, but the most striking is again the opposing impact the microRNAs have on survival for renal cancer patients.

### 2.6. Identification of Putative Repurposed Drugs Targeting PDCD1/CD274 Co-Expressed Genes/Proteins 

Due to the potential clinical relevance of the shortlisted genes and microRNAs described previously, the DRUGSURV database [25,26] was used to explore if any of the proteins generated from the genes of interest identified above were the target of an approved drug. Five proteins (tyrosine-protein kinase JAK2, CCR5, CXCR6, lymphotoxin-alpha (LTA) and mitogen-activated protein kinase kinase kinase kinase 1 (MAP4K1)) were found to be modulated by at least one drug (Table 5). The largest number of drugs was identified for the kinases, with 14 drugs targeting JAK2 and four targeting MAP4K1, followed by the chemokine receptors with four for CXCR6 and three for CCR5, with a single drug known to target the TNF-family cytokine LTA. There was some overlap in the drug targets, with one drug (disulfiram) targeting both CCR5 and CXCR6, and both sunitinib and dasatinib known to have affinity for both JAK2 and MAP4K1.

The indicated use includes a range of cancers for the drugs targeting the kinases, including cancer types used in this study. Although the drugs targeting the other proteins have not been approved for use in cancer, drugs targeting CXCR6 and/or CCR5 have been taken forward into clinical trials in the cancer setting. 

## 3. Discussion

This study utilized a bioinformatic approach to explore potential factors contributing to the PDCD1/CD274 network on a pan-cancer basis. Through this approach, several genes commonly co-expressed with PDCD1/CD274 were identified and their clinical importance ascertained through Kaplan–Meier plots. Further molecular insights were generated by assessing potential links to microRNAs, which were in turn assessed for clinical relevance. Finally, potential repurposed drugs were identified which target the genes that commonly co-express with PDCD1/CD274.

It is interesting to note that for breast cancer, renal cancer, and melanoma, high CD274 expression was statistically significantly beneficial for patients. Whilst this was initially surprising, as it is logical that CD274 expression would result in immune evasion and thereby cancer survival, a recent pooled analysis from 1062 melanoma patients showed that CD274 expression had no significant relationship with overall survival but that high CD274 expression was significantly associated with lymph node metastases [27]. As such, it is highly probable that indications of survival will be cohort specific. A similar pattern was seen for PDCD1 expression, with four of five cancers demonstrating that high PDCD1 expression was statistically significantly beneficial. Comparatively, esophageal cancer showed the opposite, with low expression being beneficial for patients (Figure 2C).

This difference could explain the starkly different Kaplan–Meier plots that were identified for the PDCD1/CD274-correlated genes, with the majority of cancers showing the same pattern for the co-expressed genes whilst esophageal and renal were notably different. Importantly, these differences could provide insight into the different underlying networks supporting the immunotherapy response and provide an opportunity for the identification of cancer type-specific adjuvants to immunotherapeutic agents.

Subsequent to the gene-level analysis was the microRNA analysis. Although 49 microRNAs were identified as linked to the CD274/PDCD1 network and many showed survival relationships (with 130 significant survival relationships being identified across fourteen cancer types), it is beyond the scope of this article to validate each of them. However, literature validation of a subset shows good consistency with previous research. For instance, a 2017 article demonstrated that hsa-miR-142-5p promotes cell growth and migration in renal cell carcinoma; therefore, low hsa-miR-142-5p expression would be beneficial for patients [28]. This is consistent with the data shown in Figure 3, where low expression of hsa-miR-142-5p was significantly beneficial for patients.

A similar outcome was that hsa-miR-337-3p was identified as potentially capable of targeting JAK2 and Table 2 demonstrates that high expression of the JAK2 gene was beneficial in several cancers, including renal and bladder cancer. In concordance with this, high expression of hsa-miR-337-3p was associated with poor outcomes in these cancer types (Figure 3). There is also experimental evidence to support the ability of this microRNA to suppress expression of JAK2 [29,30] via decreased luciferase activity upon co-transfection of the JAK2 3′UTR luciferase reporter and hsa-miR-337-3p, and decreased JAK2 protein levels upon overexpression of the microRNA in liver cancer cell lines. It should be noted, however, that the study by Zuo and colleagues [29] was conducted in hepatocellular carcinoma cells and hsa-miR-337-3p acted as a tumor suppressor and suppressed proliferation and invasion. Although Figure 3 does not demonstrate a significant survival effect for hsa-miR-337-3p in liver cancer, this points to patient cohort-specific differences and the possibility of differential survival relevance between different cancer types.

Figure 3 also shows high expression of hsa-miR-146a-5p to be beneficial in several cancers (cervical, ovarian, breast, head and neck, and gastric). The tumor-suppressive role of this microRNA is well established [31], but in other cancer types, this microRNA can act to promote cancer, working as an oncomiR [31]. Within ovarian cancer, the protective role of hsa-miR-146a-5p may be explained in part by its effect on superoxide dismutase 2 (SOD2), which leads to increased levels of reactive oxygen species (ROS), decreased proliferation, increased apoptosis, and enhanced sensitivity to chemotherapy [32].

From the panel of identified microRNAs, the one showing a significant survival association with the greatest number of cancers was hsa-miR-125-5p. The pattern was primarily for high expression of hsa-miR-125-5p to be good for patient outcomes (in pancreatic cancer, lung cancer, renal cancer, bladder cancer, and mesothelioma) which is in agreement with a recent metanalysis of patient data [33]. The mechanistic understanding of this effect is supported by a body of evidence that shows that hsa-miR-125-5p acts as a tumor suppressor in a variety of cancers. In non-small cell lung cancer [34,35] and lung adenocarcinoma [36], expression of hsa-miR-125-5p was associated with reduced cell growth, increased apoptosis, and increased differentiation. In vitro models have also identified a role for this microRNA in inhibiting invasion and migration of lung cancer cells [37]. Identified in this study as a potential modulator of DUS1L, this gene has not been experimentally verified to be the target of hsa-miR-125-5p. However, a range of identified targets are involved in the KRAS and NF-kappaB pathways, for example, SOS1, GRB2, IQGAP1, RALA, RAF-1, IKKβ, AKT2, ERK2 and KRAS [38], STAT3 [39], and antiapoptotic proteins such as BCL2, BCL2L12, MCL1 [40], BAP1 [41], and TMPRSS4 [36]. Contrary to the general trend, however, our data showed that high expression of hsa-miR-125-5p was detrimental in both gastric cancer and melanoma (Figure 3). Despite this, there is evidence in the literature that tumor expression of hsa-miR-125-5p can suppress proliferation in gastric cancer [42] and melanoma [43], together with increased senescence in melanoma [44]. This discrepancy may be explained at least in part by effects of microRNA on the tumor microenvironment. In melanoma, exosomal hsa-miR-125-5p was found to influence tumor-associated macrophages [45] and, potentially via targeting of lysosomal acid lipase A (LIPA), helps in macrophage polarization to a tumor-promoting phenotype.

A 2016 study compared microRNA expression in short-surviving and long-surviving mesothelioma patients [46] and identified that hsa-miR-17-5p, hsa-miR-22-3p, hsa-miR-27b-3p, and hsa-miR-93-5p were all expressed at a higher level in short-surviving patients versus long-surviving patients. This is partially consistent with the data presented in Figure 3, where low expression of hsa-miR-17-5p and hsa-miR-93-5p was significantly beneficial for patient survival. However, Figure 3 also shows that high expression of hsa-miR-22-3p and hsa-miR-27b-3p was beneficial for patients. Notably, the 2016 study [46] did not identify hsa-miR-1323, hsa-miR-216a-5p, hsa-miR-380-3p, hsa-miR-497-5p, hsa-miR-580-3p, hsa-miR-125b-5p, and hsa-miR-125a-5p to be important for patient survival. Differences could arise due to patient cohort-specific differences and analytical methods, as well as the number of patients included (*n* = 16 for the 2016 study [46] and *n* = 86 for the mesothelioma study from OncomiR [24]).

This study has ultimately identified a significant number of microRNAs which have prognostic importance and may be related to the molecular network of immune checkpoint blockade. Similarly, the cancer-specific differences of the co-expressed genes highlighted in Table 2 and Table 3 also warrant further investigation, particularly in the case of renal cancer which was surprisingly oppositional to the other cancer types included.

The purpose of the DRUGSURV analysis was to identify repositionable drugs that could be used to target the proteins that correlated with PDCD1/CD274. In total, 23 drugs were identified targeting various members of the network. In theory, as the genes correlate with PDCD1/CD274, targeting them in conjunction with immunotherapy could boost the response and improve outcomes, or perhaps allow for lower dosages to be used whilst maintaining therapeutic effects. Although in vitro or in vivo validation has not been performed in this study, there is literature supporting this. For example, disulfiram, targeting CCR5 and CXCR6 (Table 5), has been shown recently to have a synergistic effect with an anti-PD-1 antibody (clone J43, BioXcell ) [47]. Disulfiram, clinically approved for alcoholism, increased the numbers of cytotoxic CD8+ T cells in the tumor when combined with the anti-PD-1 antibody and ultimately inhibited tumor growth and metastasis [47]. The fact that disulfiram has been identified herein (Table 5) again provides validation to the approach utilized, and indicates that the thirteen other drugs that currently do not have a cancer indication (Table 5) could be of potential clinical utility, possibly as immunotherapeutic adjuvants.

A limitation of the present study is that only mRNA expression has been examined, and hence it is largely single-omics analyses other than the use of microRNA data in concurrence with mRNA expression. Recent reports highlight the importance of multi-omics investigation, as it allows for the flow of information at multiple biological levels to be analyzed and the data to be examined holistically [48,49,50]. That said, several promising avenues have been identified in the current study that warrant further investigation at the in vitro or in vivo level. Immunotherapy in general holds significant promise, but any clinical trial pertaining to it should interpret findings with caution, as research has shown that surrogate clinical trial endpoints such as progression-free survival are not fully reflective of overall survival and therefore caution should be used when interpreting data that do not include overall survival [51]. Ultimately, this research has identified multiple candidate genes, microRNAs, and drugs to be further investigated at the in vitro or in vivo level that could have impact in the immune checkpoint arena.

## 4. Materials and Methods

### 4.1. Study Selection

In order to interrogate the role of CD274 and PDCD1 on a pan-cancer basis, the cBioPortal database (https://www.cbioportal.org/, accessed on 29 March 2021) was accessed [19,20]. This database contains a range of patient-level multi-omics data that are easily accessible and contains data from over 300 individual studies/analyses.

To begin, to ensure consistency across the individual studies, only studies including RNA-seq data were included at the initial CD274/PDCD1 screen. Studies containing “mRNA expression z-scores relative to diploid samples (RNA Seq V2 RSEM)” for Kaplan-Meier analysis as well as “mRNA expression (RNA Seq V2 RSEM)” for co-expression were included and mapped to fifteen cancer types for which CD274/PDCD1 inhibition was an approved therapeutic strategy. This led to the studies included below in Table 6.

### 4.2. Assessing the Impact of Pan-Cancer PDCD1 and CD274 Expression 

After selecting the datasets described above in Table 6, for each cancer type in turn, all relevant studies were selected on cBioPortal and “Explore Selected Studies” was selected. Expression of CD274 and PDCD1 in turn was determined for all patients selected from each cancer type and patients were split into low and high expression based on the median value. “mRNA expression z-scores relative to diploid samples (RNA Seq V2 RSEM)” was used as the “type” of mRNA expression analysis. A Kaplan–Meier plot was then computed based on the two groups (low and high expression of CD274/PDCD1) to determine the putative role of each gene in patient survival. Raw *p* ≤ 0.05 was considered statistically significant and the determination of low or high expression being beneficial for the patient was determined by examination of the overall pattern of the curve.

### 4.3. Identifying Co-Expressed Genes/Proteins Per Cancer Type

After selecting the same studies per cancer type described in the previous section and shown in Table 6, the “Query by Gene” function on cBioPortal was utilized with CD274 or PDCD1 as an input to identify genes that co-expressed with them. “mRNA expression z-scores relative to diploid samples (RNA Seq V2 RSEM)” was again selected as the “type” of mRNA expression analysis. The default “Patient/Case Set” was used for each cancer type (though it should be noted that this defaults to “Complete samples”, meaning some patients with expression data could have been excluded). The raw co-expression outputs were downloaded and processed to filter and include correlations (Spearman’s correlation coefficient) that were ≤−0.6 or ≥0.6 (“moderately strong” [61]) per individual study with a maximum of 100 positively correlated and 100 negatively correlated per individual study. Gene lists were then collated per study for each individual cancer type.

### 4.4. Identifying Common Pan-Cancer Co-Expressed Genes 

After identifying the co-expressed genes for individual cancer studies, summary tables were created which collated the information on a pan-cancer level. Four tables in total were created: genes positively correlated with CD274; genes negatively correlated with CD274; genes positively correlated with PDCD1; and genes negatively correlated with PDCD1.

After collating the information for the summary tables, final gene lists of interest that were positively correlated with CD274 or PDCD1 were selected by filtering to include only genes that appeared in at least eight (more than half) out of fifteen cancers. For negatively correlated genes, due to the significantly fewer negative correlations identified, genes were taken forward for further analysis if they appeared in at least two different cancers. Files S1 and S2 contain the co-expression data analysis for CD274 and PDCD1, respectively. In the case of PDCD1 co-expressed genes, the list underwent a further filtering process due to the possibility of T cell-related genes appearing in the co-expression (from TILs, see File S3). This process entailed screening the function of the co-expressed genes and excluding those that appeared to be specifically T cell-related rather than tumor cell-related. The final gene lists shown in Table 1 were used to create Kaplan–Meier plots using the same criteria described previously.

### 4.5. Identifying Putative microRNAs Targeting Genes of Interest

To gain potential mechanistic insight into the CD274/PDCD1-associated genes, the miRDB database (http://mirdb.org/, accessed on 28 April 2021) was employed [22]. Genes identified from the previous section (Table 1) were entered in turn into the database to identify putative microRNAs that regulate the gene lists of interest. The miRDB site states that a prediction score > 80 is most likely to be real; therefore, 80 was used as the minimum score for the microRNA for it to be included.

Next, due to the known issue of false positives present within microRNA target prediction programs [62], a second database, miRabel (http://bioinfo.univ-rouen.fr/mirabel/, accessed 10 May 2021), was also accessed [23]. This database integrates information from miRanda [63], PITA [64], SVmicrO [65], and TargetScan [66] to provide an aggregate score with a recommended threshold of 0.05 (the lower the score the better) [23]. Genes were entered in turn into the miRabel database and microRNAs putatively targeting the genes of interest with a miRabel score ≤ 0.05 were extracted. Finally, the microRNA lists from miRDB and miRabel were cross-referenced to further reduce the likelihood of false positives, ultimately leading to the final list of microRNAs shown in Table 4. The output from each database and the cross-referencing analysis can be seen in File S4.

### 4.6. Screening the Clinical Relevance of Putative microRNAs Using OncomiR

In order to validate the clinical relevance of the identified microRNAs in Table 4, OncomiR (http://oncomir.org/, accessed on 12 May 2021) [24] was accessed. Each microRNA in turn was entered into the “Survival Outcome” section of OncomiR with *p* ≤ 0.05 used as the significance criteria. Only cancers relevant to this study were included within the results. Full OncomiR results can be seen in File S5.

### 4.7. Identification of Putative Repurposed Drugs through DRUGSURV

Following the identification of genes of interest (Table 1), each gene in turn was queried through the DRUGSURV (http://www.bioprofiling.de/GEO/DRUGSURV/index.html, accessed on 25 April 2021) database [25,26] to identify potential approved drugs that directly targeted each gene product. Links to whether the drug was indicated for a particular cancer type, or was in clinical trials, or had experimental data supporting its use, were verified using the DrugCentral (https://drugcentral.org/, accessed on 13 May 2021) database [67] and by literature searches.

## Figures and Tables

**Table 1:** Pan-cancer CD274 and PDCD1 co-expressed genes. In brackets after each gene is the number of cancers in which the gene was found to be significantly co-expressed with CD274 or PDCD1. For positive correlations, a gene was taken forward if it appeared in over half (eight of fifteen) of selected cancers. For negative correlations, due to the far smaller number of identified genes, genes were taken forward if they appeared co-expressed in more than one cancer. Genes correlated with PDCD1 underwent a further filtering process to remove co-expressed genes that appeared to be specifically T cell-related rather than tumor-cell related (File S3).

Positively Correlated with CD274	Negatively Correlated with CD274	Positively Correlated with PDCD1
PDCD1LG2 (12)	GATD3A (2)	SH2D1A (13)
GBP5 (11)	COX19 (2)	ACAP1 (12)
CD80 (9)	DNASE1 (2)	ARHGAP9 (12)
GBP1 (8)	DUS1L (2)	CXCR6 (12)
JAK2 (8)		PTPRCAP (12)
SAMD9L (8)		RASAL3 (12)
LCP2 (8)		GZMK (11)
CCR5 (8)		NKG7 (11)
		PYHIN1 (11)
		TBC1D10C (11)
		ZNF831 (11)
		CD96 (10)
		CST7 (10)
		GPR171 (10)
		GZMH (10)
		GZMM (10)
		TRAF3IP3 (10)
		GZMA (9)
		HCST (9)
		IL21R (9)
		PSTPIP1 (9)
		GIMAP5 (8)
		IKZF1 (8)
		JAKMIP1 (8)
		LTA (8)
		MAP4K1 (8)
		P2RY10 (8)
		SEPTIN1 (8)

**Table 2:** Statistically significant Kaplan–Meier results for genes correlated with CD274. It should be noted that the mesothelioma study included (Mesothelioma (TCGA, Firehose Legacy)) lacked usable Kaplan–Meier survival data.

Cancer	Positively Correlated Genes Where Low Expression Is Beneficial for Patients (*p* ≤ 0.05)	Positively Correlated Genes Where High Expression Is Beneficial for Patients (*p* ≤ 0.05)	Negatively Correlated Genes Where Low Expression Is Beneficial for Patients (*p* ≤ 0.05)	Negatively Correlated Genes Where High Expression Is Beneficial for Patients (*p* ≤ 0.05)
Cervical	N/A	N/A	N/A	N/A
Liver	N/A	CCR5	DNASE1	N/A
Ovarian	N/A	CD80	N/A	DUS1L
Esophageal	N/A	N/A	N/A	N/A
Pancreatic	N/A	N/A	N/A	GATD3A
Gastric	N/A	N/A	N/A	DUS1L
Head and Neck	N/A	CCR5	GATD3A	N/A
Lymphoma	SAMD9L	N/A	N/A	N/A
Breast	N/A	JAK2, LCP2, SAMD9L	N/A	DUS1L
Colorectal	N/A	N/A	COX19	N/A
Melanoma	N/A	CCR5, CD80, GBP1, GBP5, JAK2, LCP2, PDCD1LG2, SAMD9L	DUS1L	N/A
Lung	N/A	N/A	N/A	N/A
Renal	CD80, GBP1, LCP2	JAK2	COX19, DNASE1, DUS1L	N/A
Bladder	N/A	JAK2	N/A	DNASE1
Mesothelioma	N/A	N/A	N/A	N/A

**Table 3:** Statistically significant Kaplan–Meier results for genes positively correlated with PDCD1. It should be noted that the mesothelioma study included (Mesothelioma (TCGA, Firehose Legacy)) lacked usable Kaplan–Meier survival data.

Cancer	Positively Correlated Genes Where Low Expression Is Beneficial for Patients (*p* ≤ 0.05)	Positively Correlated Genes Where High Expression Is Beneficial for Patients (*p* ≤ 0.05)
Cervical	N/A	ACAP1, CST7, CXCR6, GPR171, GZMH, GZMK, GZMM, JAKMIP1, MAP4K1, P2YR10, PSTPIP1, RASAL3, SH2D1A, TBC1D10C, ZNF831
Liver	N/A	ACAP1, CD96, CST7, CXCR6, GIMAP5, GPR171, GZMH, GZMK, IKZF1, NKG7, P2RY10, PYHIN1, SH2D1A, TBC1D10C, TRAF3IP3, ZNF831
Ovarian	N/A	N/A
Esophageal	RASAL3	N/A
Pancreatic	N/A	SEPTIN1, ZNF831
Gastric	N/A	IL21R, JAKMIP1
Head and Neck	N/A	ACAP1, CD96, CST7, CXCR6, GPR171, GZMK, GZMM, IKZF1, IL21R, LTA, MAP4K1, NKG7, P2RY10, PTPRCAP, PYHIN1, RASAL3, SEPTIN1, SH2D1A, TBC1D10C, TRAF3IP3, ZNF831
Lymphoma	ACAP1	
Breast	N/A	ACAP1, CD96, CST7, CXCR6, GPR171, GZMA, GZMH, GZMK, GZMM, HCST, IKZF1, MAP4K1, PSTPIP1, PYHIN1, SEPTIN1, SH2D1A, TBC1D10C, TRAF3IP3, ZNF831
Colorectal	N/A	N/A
Melanoma	N/A	ACAP1, ARHGAP9, CD96, CST7, CXCR6, GIMAP5, GPR171, GZMA, GZMH, GZMK, GZMM, HCST, IKZF1, IL21R, JAKMIP1, LTA, MAP4K1, NKG7, P2RY10, PSTPIP1, PTPRCAP, PYHIN1, RASAL3, SEPTIN1, SH2D1A, TBC1D10C, TRAF3IP3, ZNF831
Lung	N/A	ARHGAP9, CXCR6, GPR171, IKZF1, LTA, MAP4K1, PSTPIP1, PTPRCAP, PYHIN1, RASAL3, SEPTIN1, TBC1D10C, TRAF3IP3
Renal	ACAP1, ARHGAP9, CXCR6, GPR171, GZMH, GZMM, HCST, IL21R, LTA, MAP4K1, PTPRCAP, RASAL3, TBC1D10C	GIMAP5
Bladder	N/A	CD96, CXCR6, GPR171, GZMA, GZMH, MAP4K1, PTPRCAP, PYHIN1, SEPTIN1, SH2D1A
Mesothelioma	N/A	N/A

**Table 4:** MicroRNAs identified in both miRDB and miRabel that putatively target CD274, PDCD1, or their correlated genes. Forty-seven of 49 microRNAs targeted a gene positively correlated with CD274/PDCD1; 2 of 49, indicated by an asterisk (*), targeted a gene negatively correlated with CD274.

microRNA Name	Gene Symbol of Target
hsa-miR-142-5p	GBP5
	ZNF831
hsa-miR-340-5p	GBP5
	ZNF831
hsa-miR-106b-5p	PDCD1LG2
hsa-miR-128-3p	SAMD9L
hsa-miR-1284	JAK2
hsa-miR-1297	GBP1
hsa-miR-1323	ZNF831
hsa-miR-135a-5p	JAK2
hsa-miR-135b-5p	JAK2
hsa-miR-146a-5p	CD80
hsa-miR-146b-5p	CD80
hsa-miR-15a-5p	CD80
hsa-miR-15b-5p	CD80
hsa-miR-16-5p	CD80
hsa-miR-17-5p	PDCD1LG2
hsa-miR-19b-3p	ZNF831
hsa-miR-204-5p	JAK2
hsa-miR-20a-5p	PDCD1LG2
hsa-miR-20b-5p	PDCD1LG2
hsa-miR-211-5p	JAK2
hsa-miR-216a-5p	JAK2
hsa-miR-218-5p	ZNF831
hsa-miR-219a-1-3p	JAK2
hsa-miR-22-3p	CD80
hsa-miR-27b-3p	TRAF3IP3
hsa-miR-302f	GBP1
hsa-miR-337-3p	JAK2
hsa-miR-377-3p	GBP1
hsa-miR-380-3p	JAK2
hsa-miR-452-5p	LCP2
hsa-miR-485-5p	ZNF831
hsa-miR-497-5p	CD80
hsa-miR-513a-5p	SAMD9L
hsa-miR-519b-3p	PDCD1LG2
hsa-miR-519c-3p	PDCD1LG2
hsa-miR-519d-3p	PDCD1LG2
hsa-miR-543	GBP1
hsa-miR-552-3p	SAMD9L
hsa-miR-561-3p	GBP5
hsa-miR-568	JAK2
hsa-miR-579-3p	LCP2
hsa-miR-580-3p	SAMD9L
hsa-miR-584-5p	GBP5
hsa-miR-629-5p	ZNF831
hsa-miR-636	ZNF831
hsa-miR-665	IKZF1
hsa-miR-93-5p	PDCD1LG2
hsa-miR-125b-5p *	DUS1L
hsa-miR-125a-5p *	DUS1L

**Table 5:** Approved drugs.

		Indication		
Drug	Target	Cancer	Other	Cancer Trial
Cytarabine	JAK2	Leukemia		Yes
Pyrimethamine	JAK2	No	Toxoplasmosis, acute malaria	Yes
Fluorouracil	JAK2	Multiple (including colon, esophageal, gastric, breast, stomach, head and neck, cervical, pancreas, renal cell)		Yes
Sunitinib	JAK2, MAP4K1	Renal cell carcinoma; gastrointestinal stromal tumor		Yes
Azathioprine	JAK2	No	Rheumatoid arthritis, transplant rejection	Yes
Floxuridine	JAK2	Liver cancer and metastases		Yes
Cladribine	JAK2	Leukemia, lymphoma		
Erlotinib	JAK2	Non-small cell lung cancer, pancreatic cancer		Yes
Albendazole	JAK2	No	Anthelmintic	
Triamterene	JAK2	No	Edema	
Podofilox	JAK2	No	Genital warts	
Dasatinib	JAK2, MAP4K1	Chronic myelogenous leukemia, acute lymphoblastic leukemia		Yes
Astemizole	JAK2	No	Allergy	
Trifluridine	JAK2	Colorectal	Keratoconjunctivitis and recurrent epithelial keratitis due to herpes simplex virus	Yes
Disulfiram	CCR5, CXCR6	No	Chronic alcoholism	Yes
Terfenadine	CCR5	No	Allergic rhinitis, hay fever, and allergic skin disorders	
Maraviroc	CCR5	No	HIV-1	Yes
Clioquinol	CXCR6	No	Antifungal	Terminated (Phase 1)
Chloroxine	CXCR6	No	Dandruff and seborrheic dermatitis	
Oxyphenbutazone	CXCR6	No		
Etanercept	LTA	No	Rheumatoid arthritis, plaque psoriasis, polyarticular idiopathic arthritis, psoriatic arthritis, ankylosing spondylitis	
Nilotinib	MAP4K1	Leukemia		Yes
Sorafenib	MAP4K1	Liver, renal		Yes

**Table 6:** Studies included from cBioPortal at the initial screening stage. N.B. Uveal melanoma was excluded from all melanoma analyses due to this cancer’s known poor response to immune checkpoint blockade compared to cutaneous melanoma [52].

Cancer Type	Study 1	Study 2	Study 3	Study 4	Study 5
Cervical	Cervical Squamous Cell Carcinoma and Endocervical Adenocarcinoma (TCGA, Firehose Legacy)				
Liver	Liver Hepatocellular Carcinoma (TCGA, Firehose Legacy)				
Ovarian	Ovarian Serous Cystadenocarcinoma (TCGA, Firehose Legacy)				
Esophageal	Esophageal Carcinoma (TCGA, Firehose Legacy)				
Pancreatic	Pancreatic Adenocarcinoma (TCGA, Firehose Legacy)				
Gastric	Stomach Adenocarcinoma (TCGA, Firehose Legacy)	Stomach Adenocarcinoma (TCGA, Nature 2014) [53]			
Head and Neck	Head and Neck Squamous Cell Carcinoma (TCGA, Firehose Legacy)	Head and Neck Squamous Cell Carcinoma (TCGA, Nature 2015) [54]			
Lymphoma	Lymphoid Neoplasm Diffuse Large B-cell Lymphoma (TCGA, Firehose Legacy)				
Breast	Breast Invasive Carcinoma (TCGA, Firehose Legacy)	Breast Invasive Carcinoma (TCGA, Cell 2015) [55]			
Colorectal	Colorectal Adenocarcinoma (TCGA, Firehose Legacy)				
Melanoma	Skin Cutaneous Melanoma (TCGA, Firehose Legacy)				
Lung	Lung Adenocarcinoma (TCGA, Firehose Legacy)	Lung Squamous Cell Carcinoma (TCGA, Firehose Legacy)	Lung Adenocarcinoma (TCGA, Nature 2014) [56]		
Renal	Kidney Renal Clear Cell Carcinoma (TCGA, Firehose Legacy)	Kidney Renal Clear Cell Carcinoma (TCGA, Nature 2013) [57]	Kidney Renal Papillary Cell Carcinoma (TCGA, Firehose Legacy)	Kidney Chromophobe (TCGA, Cancer Cell 2014) [58]	Kidney Chromophobe (TCGA, Firehose Legacy)
Bladder	Bladder Urothelial Carcinoma (TCGA, Firehose Legacy)	Bladder Cancer (TCGA, Cell 2017) [59]	Bladder Urothelial Carcinoma (TCGA, Nature 2014) [60]		
Mesothelioma	Mesothelioma (TCGA, Firehose Legacy)				

## Data Availability

All data accessed in this study are available on multiple publicly available databases as described in the Materials and Methods.

## Supplementary Materials

The following are available online at https://www.mdpi.com/article/10.3390/ijms22115478/s1.
