# Supplementary material for: Molecular Mechanisms of PD-1 and PD-L1 Activity on a Pan-Cancer Basis: A Bioinformatic Exploratory Study
Source: Int J Mol Sci. 2021 May 22;22(11):5478. doi: 10.3390/ijms22115478 (PMC8196980; doi:10.3390/ijms22115478)
Supplement: Supplementary file 1 [file ijms-22-05478-s001.zip › Supplementary Figures.pdf]

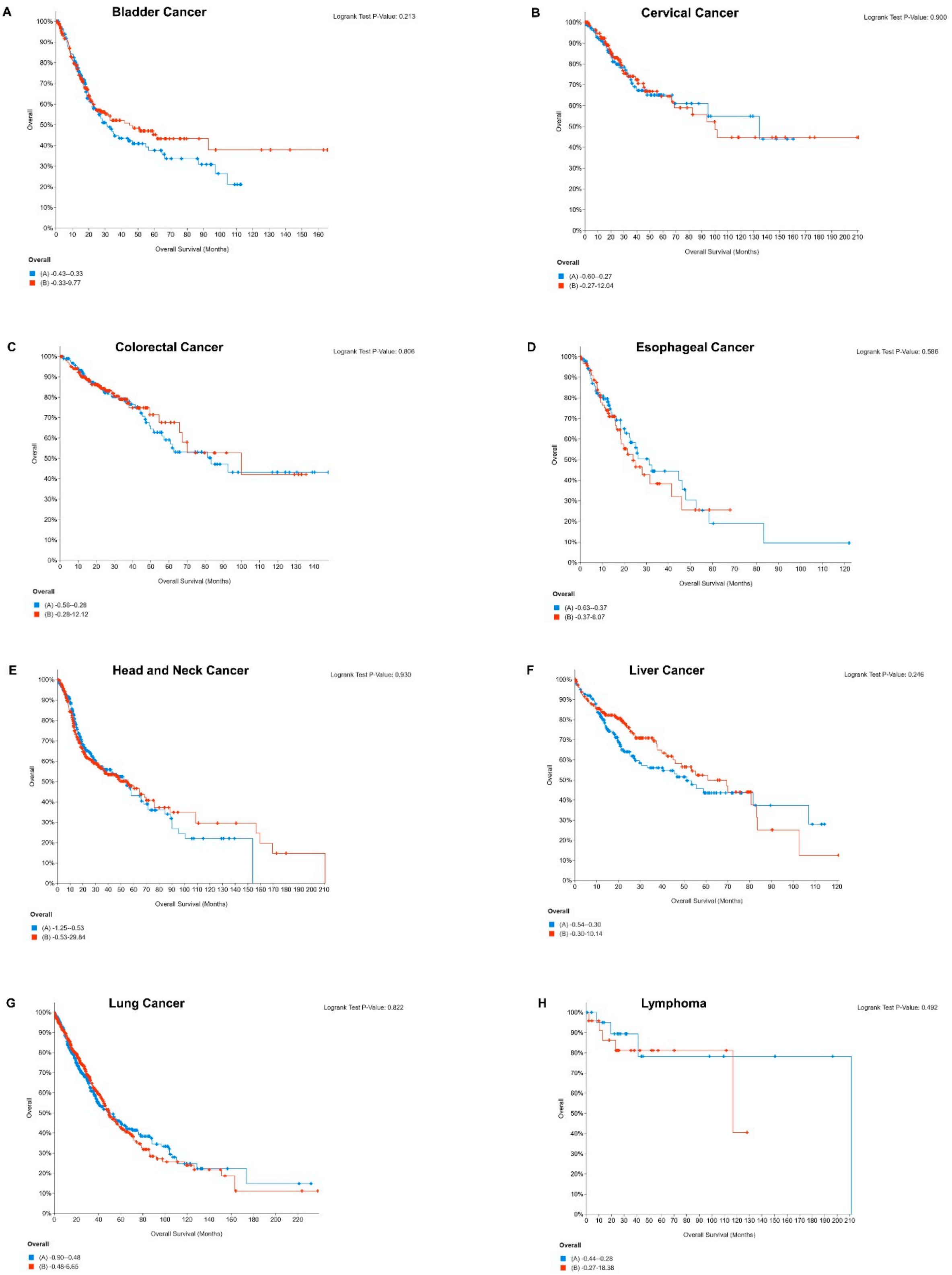
A
Bladder Cancer
Logrank Test P-Value: 0.213
Overall
Overall Survival (Months)
Overall
(A) -0.43--0.33
(B) -0.33-9.77
B
Cervical Cancer
Logrank Test P-Value: 0.900
Overall
Overall Survival (Months)
Overall
(A) -0.60--0.27
(B) -0.27-12.04
C
Colorectal Cancer
Logrank Test P-Value: 0.806
Overall
Overall Survival (Months)
Overall
(A) -0.56--0.28
(B) -0.28-12.12
D
Esophageal Cancer
Logrank Test P-Value: 0.586
Overall
Overall Survival (Months)
Overall
(A) -0.63--0.37
(B) -0.37-6.07
E
Head and Neck Cancer
Logrank Test P-Value: 0.930
Overall
Overall Survival (Months)
Overall
(A) -1.25--0.53
(B) -0.53-29.84
F
Liver Cancer
Logrank Test P-Value: 0.246
Overall
Overall Survival (Months)
Overall
(A) -0.54--0.30
(B) -0.30-10.14
G
Lung Cancer
Logrank Test P-Value: 0.822
Overall
Overall Survival (Months)
Overall
(A) -0.90--0.48
(B) -0.48-6.65
H
Lymphoma
Logrank Test P-Value: 0.492
Overall
Overall Survival (Months)
Overall
(A) -0.44--0.28
(B) -0.27-18.38

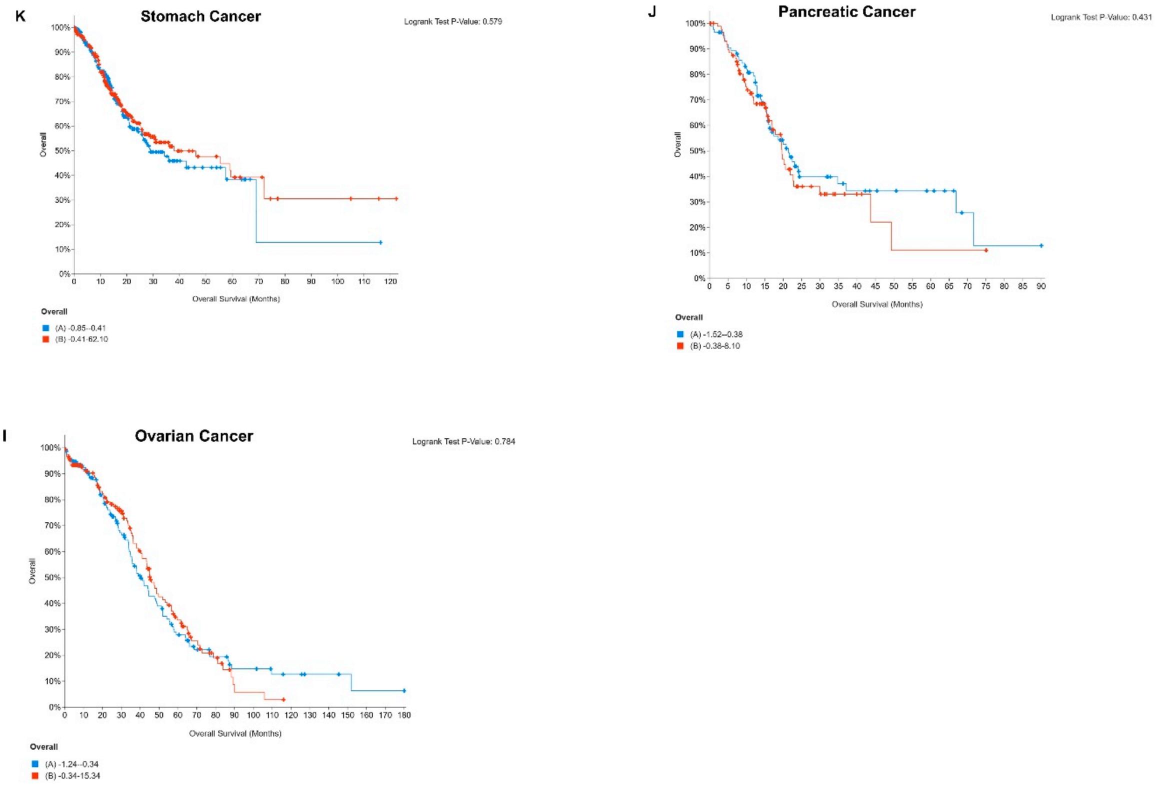

Figure S1. Non-significant Kaplan-Meier plots for CD274 expression pan-cancer. Data is shown for bladder cancer (A), cervical cancer (B), colorectal cancer (C), esophageal cancer (D), head and neck cancer (E), liver cancer (F), lung cancer (G), lymphoma (H), ovarian cancer (I), pancreatic cancer (J) and stomach cancer (K). In all cases, overall survival in months can be seen on the x-axis whilst percentage survived is shown on the y-axis. In all cases, high CD274 expression is shown in red whilst low CD274 expression is shown in blue. Data was generated using the mRNA expression z-scores relative to diploid samples (RNA Seq V2 RSEM) for the studies described in the Materials and Methods (Table 6). The mesothelioma study included (Mesothelioma (TCGA, Firehose Legacy)) lacked usable Kaplan-Meier survival data. Data for breast cancer, melanoma, and renal cancer produced significant results are shown in Figure 1.

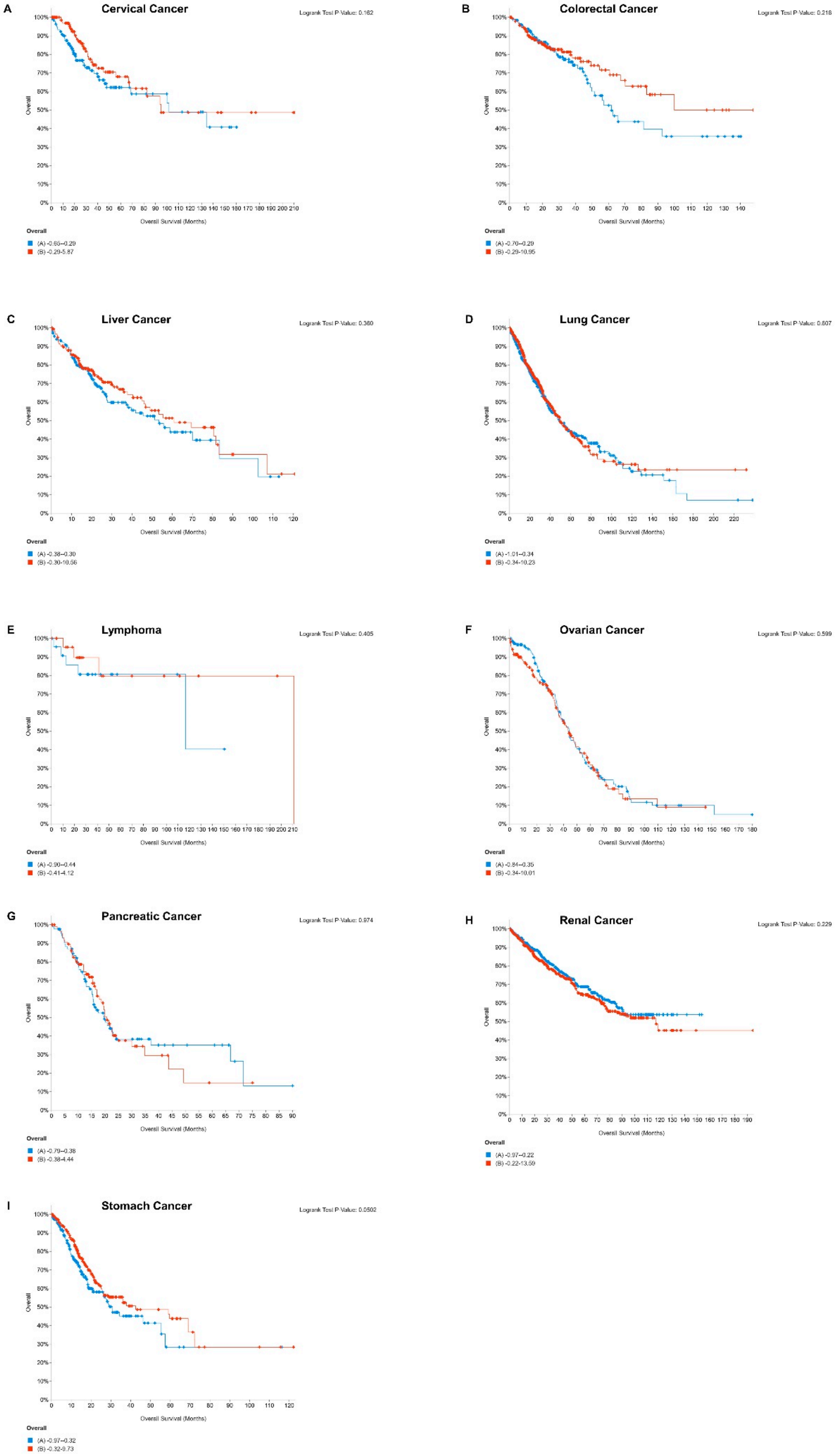
A
Cervical Cancer
Logrank Test P-Value: 0.162
Overall
Overall Survival (Months)
Overall
(A) -0.65--0.29
(B) -0.29-5.87
B
Colorectal Cancer
Logrank Test P-Value: 0.216
Overall
Overall Survival (Months)
Overall
(A) -0.70--0.29
(B) -0.29-10.95
C
Liver Cancer
Logrank Test P-Value: 0.360
Overall
Overall Survival (Months)
Overall
(A) -0.38--0.30
(B) -0.30-10.56
D
Lung Cancer
Logrank Test P-Value: 0.607
Overall
Overall Survival (Months)
Overall
(A) -1.01--0.34
(B) -0.34-10.23
E
Lymphoma
Logrank Test P-Value: 0.405
Overall
Overall Survival (Months)
Overall
(A) -0.90--0.44
(B) -0.41-4.12
F
Ovarian Cancer
Logrank Test P-Value: 0.599
Overall
Overall Survival (Months)
Overall
(A) -0.84--0.35
(B) -0.34-10.01
G
Pancreatic Cancer
Logrank Test P-Value: 0.974
Overall
Overall Survival (Months)
Overall
(A) -0.79--0.38
(B) -0.38-4.44
H
Renal Cancer
Logrank Test P-Value: 0.229
Overall
Overall Survival (Months)
Overall
(A) -0.97--0.22
(B) -0.22-13.59
I
Stomach Cancer
Logrank Test P-Value: 0.0502
Overall
Overall Survival (Months)
Overall
(A) -0.97--0.32
(B) -0.32-9.73

Figure S2. Non- significant Kaplan-Meier plots for PDCD1 expression pan-cancer. Data is shown for cervical cancer (A), colorectal cancer (B), liver cancer (C), lung cancer (D), lymphoma (E), ovarian cancer (F), pancreatic cancer (G), renal cancer (H) and stomach cancer (I). In all cases, overall survival in months can be seen on the x-axis whilst percentage survived is shown on the y-axis. In all cases, high PDCD1 expression is shown in red whilst low PDCD1 expression is shown in blue. Data was generated using the mRNA expression z-scores relative to diploid samples (RNA Seq V2 RSEM) for the studies described in the Materials and Methods (Table 6). The mesothelioma study included (Mesothelioma (TCGA, Firehose Legacy)) lacked usable Kaplan-Meier survival data. Data for breast cancer, esophageal cancer, head &neck cancer, and melanoma produced significant results are shown in Figure 2.
